# Lack of detectable DNA uptake by transformation of selected recipients in mono-associated rats

**DOI:** 10.1186/1756-0500-3-49

**Published:** 2010-03-01

**Authors:** Andrea Wilcks, Bodil BL Jacobsen

**Affiliations:** 1 Division of Microbiology and Risk Assessment, National Food Institute, Technical University of Denmark, Mørkhøj Bygade 19, DK-2860 Søborg, Denmark

## Abstract

**Background:**

An important concern revealed in the public discussion of the use of genetically modified (GM) plants for human consumption, is the potential transfer of DNA from these plants to bacteria present in the gastrointestinal tract. Especially, there is a concern that antibiotic resistance genes used for the construction of GM plants end up in pathogenic bacteria, eventually leading to untreatable disease.

**Findings:**

Three different bacterial species (*Escherichia coli*, *Bacillus subtilis, Streptococcus gordonii*), all natural inhabitants of the food and intestinal tract environment were used as recipients for uptake of DNA. As source of DNA both plasmid and genomic DNA from GM plants were used in *in vitro *and *in vivo *transformation studies. Mono-associated rats, creating a worst-case scenario, did not give rise to any detectable transfer of DNA.

**Conclusion:**

Although we were unable to detect any transformation events in our experiment, it cannot be ruled out that this could happen in the GI tract. However, since several steps are required before expression of plant-derived DNA in intestinal bacteria, we believe this is unlikely, and antibiotic resistance development in this environment is more in danger by the massive use of antibiotics than the consumption of GM food harbouring antibiotic resistance genes.

## Findings

A major concern in relation to marketing of genetically modified (GM) plants for human consumption is the possible transfer of antibiotic resistance genes used as marker genes in GM plants to the human or animal intestinal microbiota. The uptake of these resistance genes by bacteria present in the gastrointestinal (GI) tract could potentially render pathogens resistant to antimicrobial agents currently used, thereby resulting in untreatable diseases [1].

Transformation is the only known gene transfer mechanism by which bacteria can take up DNA released from plants. Key factors are thus DNA persistence in the GI tract, the availability of competent bacteria, and their state of competence [2]. Several studies indicate that DNA, and especially plant-associated DNA, is able to survive the conditions in the GI tract and be available for uptake by bacteria resident in the gastrointestinal tract [3-8].

Several of the bacteria found in the GI tract, either carried by the food or innate GI bacteria, have been found to be naturally transformable [9]. But the question is whether these bacteria also possess or develop competence in this environment. In this work we used as recipients the naturally transformable bacteria *Bacillus subtilis *which is often a contaminant of food, and *Escherichia coli *and *Streptococcus gordonii *that are part of the normal gut microbiota. We used mono-associated rats that can be considered as a worst-case model, and as a biological magnifier making it possible to study one bacterial species separately and often in high number. All animal experiments were carried out under the supervision of the Danish National Agency for Protection of Experimental Animals.

### *Escherichia coli*

*In vitro *experiments were performed using an overnight culture of DB1317 (Table 1) mixed with plasmid pMR2 (100 μg/ml) in Luria-Bertani (LB) media and incubated at 37°C. Sampling at 2 and 4 hrs gave rise to transformants on LB media containing chloramphenicol (25 μg/ml) at a frequency of up to 10^-7 ^transformants (TF) per recipient (data not shown). Plasmid extraction and restriction analysis confirmed that transformants harboured the plasmid pMR2 (data not shown). Cells incubated without addition of plasmid DNA (negative controls) did not give rise to any chloramphenicol resistant colonies. When adding faecal or intestinal samples from germfree rats to the LB medium to a final concentration of 10%, and performing the same experiment as described above, no transformants were detected (detection limit 2.3 × 10^-9 ^TF/rec).

**Table 1:** Bacterial strains and plasmids

Strains or plasmids	Description	Reference or source
Bacterial strains:		

*Escherichia coli*		

DB1317	*recD1014 *(Nuc^-^)	CGSC

MS15978	DB1317 harbouring pMR1, Ap^r^	This study

MS14395	DH5α, *recA1*, harbouring pMR2, Cm^r^	[14], this study

*Bacillus subtilis*		

Strain 168	1A700	BGSC

*Streptococcus gordonii*		

LTH 5597	TIGR strain harbouring pMK110, Ery^r^	[12]

Plasmids:		

pMR1	pBR322 vector, *nptIIΔNco*I, Ap^r^	[10]

pMR2	pACYC184 vector, *nptII *without promoter, Cm^r^	[10]

pMK110	pMG36e vector, *nptIIΔNco*I from pMR1, Ery^r^	[15]

pAW105	pUC19 vector, *cat *from pC194, *Bacillus thuringiensis *replicon, Ap^r^	[16]

Plant:		

*Solanum tuberosum *cv. Apriori	Genetically modified potato containing antisense *GBSS *(granule bound starch synthase) and intact *nptII*	AVEBE, Foxhole, The Netherlands

Abbreviations: Ap^r^: ampicillin resistance; BGSC: Bacillus Genetic Stock Center; *cat*: chloramphenicol acetyltransferase gene; CGSC: *E. coli *Genetic Stock Center; Cm^r^: chloramphenicol resistance; Ery^r^: erythromycin resistance; GBSS: granule bound starch synthase gene;*nptII*: kanamycin resistance gene.

**Figure 1:**
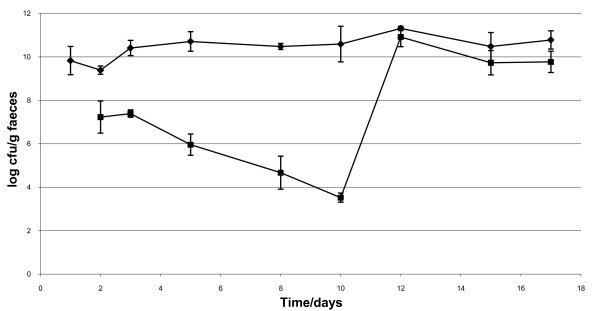
**Colonization of four mono-associated rats with *E. coli *strain MS15978**. Faecal samples were plated on both BHI (◆) and BHI including ampicillin (■). From day 10 all rats were dosed with 1000 μg ampicillin each day to restore the population of pMR1 containing *E. coli *cells. The detection limit for detection of transformants was 100 cfu/g faeces. Vertical bars represent standard error of the mean (SEM).

Although *ex vivo *experiments with intestinal and faecal samples could not detect any uptake of DNA, the situation may be different *in vivo *where specific environmental factors may be present enhancing uptake of DNA by *E. coli*. Therefore an *in vivo *experiment with *E. coli *strain MS15978 (DB1317 harbouring plasmid pMR1) was performed. Four germfree Sprague-Dawley rats (14-17 weeks old) bred and housed as previously described [6] were dosed p.o. by gavage with 1 ml cell suspension (app. 10^9 ^cfu/ml) of *E. coli *strain MS15978 at day 1 of the experiment. One rat served as a control and was not fed with plasmid DNA, while the other three animals received 100 μg plasmid pMR2 each working day from day 8 to 17 of the experiment. Dosing the rats with plasmid pMR2 gave us the opportunity to look at uptake (chloramphenicol resistance, 25 μg/ml) as well as homologous recombination (kanamycin resistance, 500 μg/ml) by marker-rescue of the deleted *nptII *gene on pMR1 [10]. The strain colonized the animals to a concentration of up to 10^11 ^cfu/g faeces (Figure 1). However, the plasmid pMR1 was not stably maintained in the strain MS15978 during colonization of the animals. Therefore a selective pressure was put on the plasmid (1000 μg/rat/day of ampicillin p.o. by gavage) at day 10 to 17 of the experiment. This restored the population of pMR1 containing cells (see Figure 1). At sacrifice at day 17, pMR1 containing *E. coli *cells were found throughout the intestinal tract at the following app. densities: duodenum: 10^3 ^cfu/g; ileum: 10^6 ^cfu/g; caecum and colon: 10^8 ^cfu/g. Transformants were detected neither in faecal or intestinal samples on Brain Heart Infusion (BHI) media containing either chloramphenicol (25 μg/ml) or kanamycin (500 μg/ml) (detection limit 100 cfu/g).

In this study we observed low-frequency spontaneous transformation of *E. coli *strain DB1317 growing in LB media without addition of divalent cations or temperature shift, conditions pivotal for high frequency transformation. However, adding faecal or intestinal samples from germfree rats to the LB media let drop the number below the detection limit of 2.3 × 10^-9 ^TF/rec. This could indicate that adding these samples either inhibited uptake of DNA, or that the DNA was degraded in the samples. A recent published paper (Nordgaard et al., 2007) observed the same inhibitory effect of intestinal content from germfree mice on the transformation process of *Acinetobacter baylyi*. Previous studies incubating plant DNA in GI samples *ex vivo *showed that the DNA was rapidly degraded in samples from the small intestine, whereas hardly any degradation was observed when incubating the DNA in samples from the lower part of the GI tract [6]. However, DNA persistence was studied using PCR, a sensitive method needing only scarce amounts of DNA for amplification. Studies incubating plasmid DNA in faecal samples from germfree rats showed that by simple gel electrophoresis, the plasmid could not be observed after 10 minutes of incubation, whereas by PCR a strong band could be detected even after 20 min of incubation (unpublished results). Since the transformation frequency in LB media is already low, the degradation of DNA in the samples is probably lowering the transformation frequency further, so that a possible transfer event is below detection limit. The same may be true for the *in vivo *studies where no uptake of DNA by *E. coli *could be observed, in concordance with an earlier study showing that although plasmid DNA was detected in the GI tract, the concentration was very low [6].

### *Bacillus subtilis*

**Figure 2:**
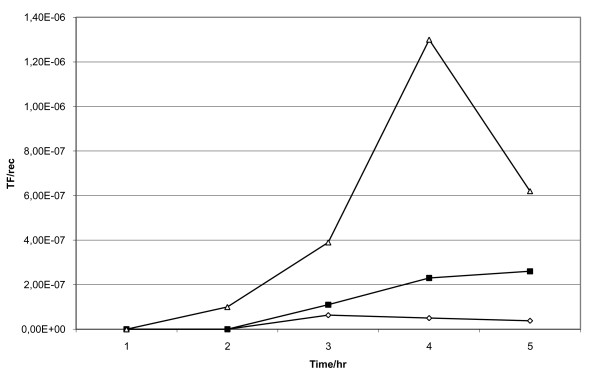
***In vitro *transformation frequencies (transformants per recipient) of *B. subtilis *168**. An overnight culture was diluted in MS media to OD_450 _= 0.25. The culture was grown under aerobic conditions at 37°C. Every hour, one ml was taken out and different amounts of plasmid (0.1 μg/ml [Δ], 1 μg/ml [◆], and 10 μg/ml [■] were added. The cultures were incubated for further 1 hr before plating on selective media for counting of recipients (LB) and transformants (LB + 5 μg/ml chloramphenicol).

Using minimal salt (MS) medium, the plasmid pAW105, and following the *in vitro *transformation procedure of Spizizen [11] we got a transformation frequency of up to 10^-6 ^TF/recipient (Figure 2). PCR with primers detecting pAW105 sequences confirmed that the obtained transformants harboured the plasmid (data not shown). As such the used system for *in vitro *studies can be regarded as an efficient transformation system. Supplementing the MS medium with 10% intestinal contents (stomach, ileum, caecum, or colon) from *B. subtilis *mono-associated rats resulted in transformants from all samples with a frequency of up to 1.1 × 10^-7 ^TF/recipients (data not shown). This showed that this concentration of intestinal content allows transformation of *B. subtilis *to nearly the same extent as in pure MS medium.

**Figure 3:**
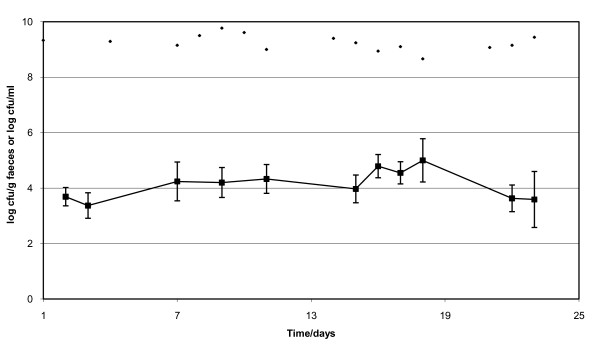
**Faecal concentration (cfu/g faeces) of *B. subtilis *recipients (■) in eight mono-associated rats**. The concentration of the dosage culture (cfu/ml) given during the three weeks is also indicated (◆). The detection limit for detecting transformants was 10 cfu/g faeces. Vertical bars represent SEM.

One animal experiment was performed with ten germfree rats mono-associated with *B. subtilis *168 and eight of those fed high amounts of plasmid pAW105. Figure 3 shows that in spite of daily doses of overnight cultures containing app. 10^9 ^*B. subtilis *cells, the bacteria only reached a concentration in faeces ranging from 10^3 ^to 10^5 ^cfu/g. During the experimental period of three weeks, the animals received about 100 μg of plasmid DNA each working day, but neither in faecal or intestinal samples transformants on LB + Cm (5 μg/ml) were detected (detection limit 10 cfu/g).

An explanation of lack of transformants in the rats could be the low number of *B. subtilis *present in the GI tract with app. 10^3 ^cfu/g in ileum, 10^3 ^cfu/g in caecum and 10^5 ^cfu/g in colon. *In vitro *the highest transformation frequency observed was 10^-6 ^TF/recipient. Therefore the likelihood of detection of a transformed *B. subtilis *within a faecal population of 10^3^-10^5 ^cfu/g is very low.

### *Streptococcus gordonii*

Previous published studies have shown that the used strain, LTH 5597, is capable of taking up plasmid DNA and genomic plant DNA under *in vitro *conditions [12].

**Figure 4:**
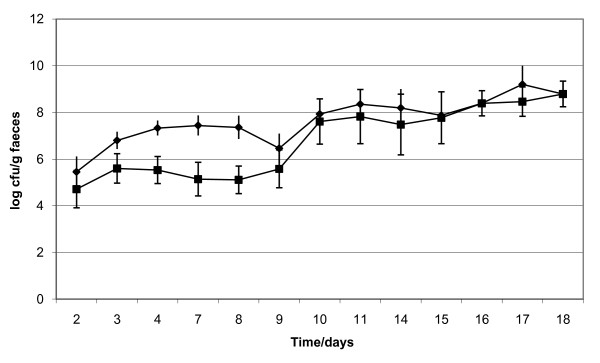
**Colonization of eight animals with *S. gordonii *LTH 5597**. Faecal samples were plated on both BHI (◆) and BHI including erythromycin (■). From day eight, all rats were dosed with 100 μg erythromycin each day to restore the population of pMK110 containing cells. The detection limit for detection of transformants was 10 cfu/g faeces. Vertical bars represent SEM.

The strain LTH 5597 given by p.o gavage to ten germ-free rats at day 1 and 4 colonized well (Figure 4), but it was noticed that the amount of erythromycin resistant cells was declining during the first week, indicating that plasmid pMK110 was lost from the population. Therefore, from day 8 of the experiment all animals received 100 μg erythromycin each working day throughout the rest of the experiment. As can be seen from the figure this restored the population of cells containing plasmid pMK110.

At day 7 to 10 of the experiment, eight animals received 1 mg DNA extracted from GM potato; this corresponds to approximately 10^9 ^*nptII *genes. Faecal samples were taken and plated onto selective media (BHI containing 1 mg/ml kanamycin), but no transformants were detected (detection limit 10 cfu/g faeces). To select for potential transformants, samples were pooled, incubated in Brain Heart Infusion (BHI) media containing 1 mg/ml kanamycin at 37°C overnight, and plated onto selective media containing kanamycin. But again no transformants were detected (detection limit 10 cfu/ml).

At day 22 of the experiment, the test animals received an overnight culture of *E. coli *harbouring the plasmid pMR2, which contains a whole copy of *nptII *but without a promoter. The strain established well in the animals with app. 10^8 ^cfu/g faeces, but again no kanamycin resistant colonies of *S. gordonii *were detected (detection limit 10 cfu/g faeces). At sacrifice the total number of bacteria in the different sections of the intestine where as following: duodenum: 10^4 ^cfu/g; ileum: 10^6 ^cfu/g; caecum and colon: 10^8 ^cfu/g. And also here no transformants were detected (detection limit 10 cfu/g intestinal content). This is in agreement with another study with germ free rats mono-associated with *S. gordonii *and fed large amounts of plasmid DNA that also failed to show transformation *in vivo *[12].

## Concluding remarks

In the present study, the three studied bacterial species were unable to take up free DNA in a germfree animal model. Using germfree mice, another study using *Acinetobacter baylyi *also failed to show *in vivo *transformation [13]. However, there are several more transformable bacterial species that are relevant for the intestinal situation [9], so we cannot rule out the possibility of transformation to happen in the GI tract. Nevertheless, many steps are required before a successful transfer of an antibiotic resistance gene from plant to bacteria has occurred. The DNA has to be released from the plant material, the DNA must survive the harsh gastrointestinal environment; competent bacteria must be present and take up the DNA, and finally has the gene to be integrated into the genome in a place where it can be expressed. Therefore the development of antibiotic resistant pathogenic bacteria is much more favoured by the use of antibiotics thereby putting a selective pressure on the intestinal environment, than by the consumption of antibiotic resistance genes present in GM plants.

## Competing interests

The authors declare that they have no competing interests.

## Authors' contributions

AW participated in the design of the study, carried out the microbial and molecular work, and drafted the manuscript. BJ conceived of the project and participated in the design of the animal studies. Both authors read and approved the final manuscript.
